# Extensive deep vein thrombosis following prolonged gaming (‘gamer’s thrombosis’): a case report

**DOI:** 10.1186/1752-1947-7-235

**Published:** 2013-10-08

**Authors:** Hsien-Cheng Leon Chang, Hayley Burbridge, Conroy Wong

**Affiliations:** 1 Department of Medicine, Middlemore Hospital, 100 Hospital Road, Otahuhu, Auckland 2025, New Zealand

**Keywords:** Video games, Venous thromboembolism, DVT

## Abstract

**Introduction:**

The average time spent playing video games is increasing. Prolonged immobility associated with gaming may therefore be an important risk factor for venous thromboembolism. We report a case of deep vein thrombosis associated with prolonged playing of PlayStation^®^ games.

**Case presentation:**

A 31-year-old Caucasian man, an exterior painter, presented with a three-day history of left leg pain and swelling after playing PlayStation^®^ games for almost eight hours a day for four consecutive days. Doppler ultrasound of the left leg confirmed extensive left leg deep venous thrombosis requiring thrombolysis and anticoagulation.

**Conclusions:**

Video gaming should be considered a risk factor for venous thromboembolism. Further studies are needed to estimate the degree of risk associated with prolonged periods of playing video games, and education for preventing venous thrombosis should be provided to gamers.

## Introduction

A period of prolonged seated immobility is recognised as one of the major risk factors for developing venous thrombosis. Long-distance air travel and prolonged sitting in relation to work or recreation have been shown to increase the risk of venous thrombosis [1,2]. A recent survey has found that the average time spent playing video games is increasing and that gamers in the United States spend an average of 13 hours each week playing computer games [3]. Prolonged immobility associated with gaming may therefore be an important risk factor for venous thromboembolism (VTE). We report a case of a 31-year-old man who developed extensive deep vein thrombosis (DVT) associated with prolonged playing of PlayStation^®^ games.

## Case presentation

A 31-year-old Caucasian man, an exterior painter, presented with left leg pain and swelling. He was on holiday and spent each day sitting on his bed with his legs outstretched playing PlayStation^®^ games. He would play for seven to eight hours continuously without getting off the bed. On the second day, he developed left leg pain with associated calf swelling and erythema, but, despite increasing discomfort, continued to play video games until presenting two days later. There was no history of trauma to his left leg, recent surgery, or previous venous thrombosis. There was no family history of venous thrombosis. On examination there was marked swelling of the leg with dilated superficial veins. Doppler ultrasound of his left leg confirmed extensive deep venous thrombosis involving the origin of the left common iliac vein down to level of the distal left femoral vein and proximal long saphenous vein, with features indicating complete occlusion. He was commenced on anticoagulation with subcutaneous enoxaparin, and subsequently underwent thrombolysis with heparin and urokinase. A thrombophilia screen was negative.

## Discussion

The pathophysiology of venous thrombosis involves venous stasis, hypercoagulability, and endothelial dysfunction, which was termed Virchow’s triad, described initially in 1856 by the German pathologist Ruldoph Virchow [4]. The association between prolonged sitting and venous thromboembolism (VTE) was first recognised by Simpson during the London Blitz in World War II, when he reported deaths from pulmonary embolism in people spending hours sitting in chairs in air-raid shelters [5]. Today, prolonged air travel is well recognised as an important risk factor for venous thrombosis [6], a phenomenon called ‘economy class syndrome’ [1]. Increasing attention is now being paid to the potential risk imposed by a more sedentary lifestyle that has developed over recent decades in relation to occupation and recreation. Beasley *et al*. used the term ‘eThrombosis’ to describe the 21st century variant of venous thrombosis and reported a case of life-threatening VTE in association with prolonged immobility sitting at a computer [5]. The authors later renamed the condition the ‘seated immobility thromboembolism’ (SIT) syndrome in view of the different occupations and recreations associated with seated immobility [2]. One case–control study showed that prolonged work- and computer-related seated immobility, which was defined as being seated at work and on the computer at home for at least 10 hours in a 24-hour period and for at least two hours at a time without getting up, was associated with a 2.8-fold increased risk of VTE [7].

There has been a growing popularity of video games over the last 30 years, with annual sales in the United States of more than $11 billion. The age of gamers has increased and in 2012, 37 percent of American video game players were older than 36 years of age [8]. The time spent on gaming has also increased. The risk of venous thrombosis is likely to be higher in extreme gamers, who represent 4 percent of the total US gaming population and spend 48.5 hours a week playing games [3]. A case was reported in 2004 where a 24-year-old died from pulmonary embolism (PE) following approximately 80 hours of continuous play [9]. Video gaming should be considered as part of the risk assessment of venous thromboembolism. Those at risk could be advised about regular leg exercises, adequate hydration and regular breaks [10].

While prolonged seated immobility is a risk factor for the development of venous thrombosis, there could be other factors related to prolonged gaming that are involved in the pathogenesis of VTE. Previous research has shown an increase in blood pressure and heart rate with exposure to violent video games as part of the physiological stress response [11], suggesting an association between acute psychological stress and a hypercoagulable state [12,13]. The prolonged period of mental stress associated with video gaming could further increase the risk of venous thrombosis in the setting of seated immobility.

## Conclusions

With the growing popularity of video games, the burden of this presentation of venous thrombosis is likely to increase. Further studies are needed to estimate the degree of risk associated with prolonged periods of playing video games, and education for preventing venous thrombosis should be provided to gamers.

## Consent

Written informed consent was obtained from the patient for publication of this case report and accompanying images. A copy of the written consent is available for review by the Editor-in-Chief of this journal.

## Competing interests

The authors declare that they have no competing interests.

## Authors’ contributions

LC participated in writing and editing the report. CW and HB participated in editing the report. All authors read and approved the final manuscript.
